# MiRNA155HG polymorphisms influenced the risk of liver cancer among the Han Chinese population

**DOI:** 10.1186/s12881-020-01064-4

**Published:** 2020-06-19

**Authors:** Xu Chao, Xuesong Feng, Xiaoping Wang, Hailong Shi, Hong Li, Yuewen Wang, Lanlan Wang, Haiyu Shen, Qing Zha, Yanni Chen

**Affiliations:** 1 The College of Basic Medicine, The Shaanxi University of Chinese Medicine, Xianyang, 712046 Shaanxi China; 2 The Second Affiliated Hospital of Shaanxi University of Chinese Medicine, Xianyang, 712000 Shaanxi China

**Keywords:** Liver cancer, *MIR155HG*, Single nucleotide polymorphisms (SNPs), Case-control study

## Abstract

**Background:**

Liver cancer is one of the most common cancers in the world. The primary aim of this research was to discover the correlation between single nucleotide polymorphisms (SNPs) of the *MIR155HG* and liver cancer risk.

**Methods:**

The selected SNPs in *MIR155HG* were genotyped utilizing the Agena MassARRAY platform. We evaluated the correlation between *MIR155HG* polymorphisms and Liver cancer by genetic model analysis, stratification analysis and haplotype analysis. Relative risk of Liver cancer was shown based on odds ratios (ORs) and 95% confidence intervals (95% CIs).

**Results:**

Our results uncovered that rs12482371 and rs1893650 in the *MIR155HG* were associated with protection against Liver cancer. And the rs928883 was related to increase risk of Liver cancer. Furthermore, apart from rs77218221, other selected SNPs formed two LD blocks, and haplotype “GATAG” in block 2 elevated individual liver cancer risk.

**Conclusions:**

*MIR155HG* gene polymorphism may be correlated to Liver cancer susceptibility in Han Chinese population, particularly in males and aged ≤55 years.

## Background

Globally, Liver cancer is a common malignancy and the second leading cause of cancer death worldwide [1], accounting for 466,100 new patients and 422,100 deaths per year in China [2]. The major type of Liver cancer (70-80%) is hepatocellular carcinoma (HCC), followed by intrahepatic cholangio carcinoma [3]. Among all cancers in China, liver cancer had poorest survival with a 5-year relative survival rate of only 10.1% [4]. Due to the difficulty of early diagnosis, most of the patients are identified at an advanced stage with numerous lesions and extrahepatic metastasis. Moreover, as a highly aggressive malignancy with rapid progression, Liver cancer is difficult to control because of low selectivity and limited chemotherapeutic drugs [5]. So there is urgent need to better understand the potential mechanism of cancer development, especially in Chinese people. Liver cancer is a complex process, associated with many factors and co-factors. Hepatitis B virus (HBV) is commonly accepted to contribute to be the leading cause of Liver cancer [6, 7]. Occupational and environmental factors, such as certain metabolic Liver diseases and cirrhosis, have also been reported to contribute to the incidence of Liver cancer [8]. In addition, similarly with the breast cancer and glioma [9, 10], the occurrence of Liver cancer is also influenced by genetic factors, and the associations have been proved in many studies [11–13]. For example, Kumar V et al. found that genetic variant rs2596542 located in the 5′ flanking region of MICA was strongly linked to HCV-induced hepatocellular carcinoma [14]. Zhang H et al. identified that internal polymorphism locus rs17401966 in KIF1B gene was highly correlated with HBV- associated HCC [15].

Long noncoding RNAs (lncRNAs) have no coding protein potential with a length of more than 200 nucleotides. They have been mistaken for genetic dark matter for a Long time. However, in recent years, studies have confirmed that such RNAs participate in a variety of regulation in biological activities, such as transcriptional inhibition and post-transcriptional regulation [16]. *MIR155HG* is a typical lncRNA, known as B-cell Integration Cluster (*BIC*) [17]. This gene located on chromosome 21 of human. Tam subsequently proved that it is encoded in a highly conserved region of the third exon of the BIC [18]. In addition, miR-155 expression is closely related to tumor. Xuechao Wu et al. revealed that *MIR155HG* can promote the development of glioma, and showed cancer-promoting activity by expressing miR-155-5p/−3p [19]. Siqi Chen et al. discovered that host miR-155 activates tumor growth via a myeloid-derived mechanism of inhibiting cell-dependence [20]. Given the importance of *MIR155HG* in glioma, we hypothesized that germline genetic variants within *MIR155HG* would influence the course of Liver cancer. As the most common genetic variation, single nucleotide polymorphisms (SNPs) can influence the development of diseases by influencing gene expression and its function. Therefore, we conducted a case-control study to discover the effects of genetic polymorphisms in *MIR155HG* on liver cancer susceptibility in Han Chinese population.

## Methods

### Study participants

A total of 432 liver cancer cases were recruited from the Shaanxi University of Chinese Medicine from August 2017 to December 2018. The inclusion criteria for eligible case were newly diagnosed primary liver cancer patients and histologically confirmed by at least two pathologists. The exclusion criteria for patients were family history of non-liver cancer, or any other malignancy and digestive diseases, such as hepatitis and cirrhosis of the liver. None of the patients received any treatment before blood collection. During the same period, 430 healthy controls were randomly enrolled from the same hospital’s physical examination center. The exclusion criteria of controls were family history of liver cancer more than three generations and chronic digestive disease. All participants were Han Chinese and there was no blood relationship between each participant.

### DNA extraction

Blood samples from each participant were drawn and stored with ethylenediamine tetraacetic acid (EDTA) at − 80 °C. Genomic DNA was extracted from peripheral blood using the GoldMag extraction method (GoldMag Co Ltd., Xi’an, China). The quality of DNA samples was detected by the NanoDrop 2000 (Thermo Scientifc, Waltham, Massachusetts, USA).

### SNPs selection and genotyping

The genetic information of the SNPs was obtained from dbSNP database and 1000 Genomes database (http://www.internationalgenome.org/). Only the variant with minor allele frequency (MAFs) > 5% in Asian population are eligible in this study in order to achieve adequate statistical power. Therefore, we finally picked up eight candidate polymorphisms (rs4143370, rs77218221, rs12482371, rs77699734, rs11911469, rs1893650, rs34904192, and rs928883) to perform analysis in Chinese Han population. According to Agena online software, the primers for the eight related sites were designed (Supplementary Table 1). SNPs were genotyped by Agena MassARRAY RS1000 (Agena, San Diego, CA, U.S.A.) (Supplementary Table 2) [21].

### Statistical analysis

We applied SPSS version 19.0 (SPSS, Chicago, IL, USA) and Microsoft Excel software for statistical analysis. Hardy-Weinberg equilibrium in controls was determined via χ^2^ test [22]. The distribution differences were evaluated using the chi-square test for sex and independent sample student’s t test for age between cases and controls. The odds ratio (OR) values and 95% confidence intervals (CIs) were calculated to assess the relationships between *MIR155HG* polymorphisms and liver cancer risk [23]. The influence of SNPs in the *MIR155H* on individual liver cancer risk was assessed by four genetic model analyses (genotype, dominant, recessive and log-additive) through PLINK software (Version 1.07). Further, we conducted stratification analysis by both age and sex. Then, statistical power of each SNP was examined through the online software (http://sampsize.sourceforge.net/iface/s3.html#ccp) in our research. Finally, the Haploview software package (version 4.2) was used to assess the linkage disequilibrium (LD) and performed haplotype analysis.

## Results

### Characteristics of the participants

We selected 432 liver cancer cases and 430 healthy controls in this research. The case group included 344 males and 88 females. The control group included 342 males and 88 females. The average age among cases and controls was 55.09 ± 11.59 years and 55.22 ± 10.73 years, respectively. The demographic characteristics of the case and control groups were listed in Table 1. The statistical analysis results manifested that age and gender between two groups had no statistically significant difference (*p* > .05).

**Table 1:** Characteristics of patients with liver cancer and controls

Characteristics	Cases *n =* 432	Controls *n =* 430	*p-*value
Age, years	55.09 ± 11.59	55.22 ± 10.73	0.972^a^
> 55	209	185	
≤ 55	223	245	
Gender			0.861^b^
Male	344	342	
Female	88	88	

^a^*p* values were calculated by Student’s t tests

^b^*p* values were calculated from two-sided chi-square tests

### The associations between MIR155HG and liver cancer

Basic information of candidate SNPs in the *MIR155HG* were listed in Table 2, including chromosomal position, allele, minimum allele frequency (MAF), HWE test results and the *p* value of allele model analysis. All the selected SNPs met HWE (*p* > 0.05). We evaluated the risk of gene polymorphism in the allele model by chi-squared test, and found that there were no associations between the SNPs and the risk of Liver cancer with all *p* > 0.05.

**Table 2:** Basic information about the candidate SNPs in this study

SNP ID	Chr.Position	Alleles	MAF		*p*-HWE	OR (95%CI)	*p*-value
		(minor/major)	Cases	Controls			
rs4143370	21:25564661	C/G	0.169	0.154	0.715	1.12 (0.86 - 1.44)	0.407
rs77218221	21:25565063	C/T	0.047	0.046	0.613	1.02 (0.66 - 1.60)	0.918
rs12482371	21:25566041	C/T	0.284	0.317	0.095	0.85 (0.69 - 1.05)	0.125
rs77699734	21:25566995	C/G	0.074	0.095	0.784	0.76 (0.54 - 1.07)	0.120
rs11911469	21:25567971	A/C	0.122	0.107	0.804	1.16 (0.86 - 1.56)	0.334
rs1893650	21:25568503	C/T	0.182	0.196	0.879	0.91 (0.72 - 1.16)	0.447
rs34904192	21:25569623	A/G	0.238	0.248	0.438	0.95 (0.76 - 1.18)	0.634
rs928883	21:25571713	A/G	0.494	0.454	0.624	1.18 (0.97 - 1.42)	0.099

*CI* confidence interval, *HWE* Hardy–Weinberg equilibrium, *MAF* minor allele frequency; *p*-HWE < 0.01 indicates imbalance, *OR* odds ratio, *SNP* single-nucleotide polymorphism

*p < 0.05 indicates statistical significance

*p < 0.00625 (0.05/8) indicates statistical significance for Bonferroni correction

As shown in Table 3, four genetic models (codominant, dominant, recessive, and additive genetic models) were applied to analyze the correlation between SNPs and liver cancer. For each variation, the minor allele was a risk factor when compared to the wild-type (major) allele in the genetic model. Our results found that rs12482371 in *MIR155HG* was associated with a 0.56-fold decrease in risk for Liver cancer under genotype model (adjusted, OR = 0.56, *p* = 0.022 for the “C/C” genotype) and a 0.55-fold decreased in risk for Liver cancer under recessive model (adjusted, OR = 0.55, *p* = 0.015 for the “C/C” genotype). The rs1893650 in *MIR155HG* was associated with a 0.35-fold decrease in risk for liver cancer under genotype model (adjusted, OR = 0.35, *p* = 0.030 for the “C/C” genotype) and a 0.34-fold decreased in risk for Liver cancer under recessive model (adjusted, OR = 0.34, *p* = 0.026 for the “C/C” genotype). The rs928883 in *MIR155HG* was associated with a 1.71-fold increase in risk for Liver cancer under genotype model (adjusted, OR = 1.71, *p* = 0.001 for the “A/G” genotype) and a 1.60-fold increase in risk for Liver cancer under dominant model (adjusted, OR = 1.60, *p* = 0.003 for the “A/G-A/A” genotype). We have found no other SNPs on *MIR155HG* associated with the susceptibility to Liver cancer risk, either uncorrected or adjusted for age and sex.

**Table 3:** Relationships between *MIR155HG* polymorphism and liver cancer risk

SNP ID	Model	Genotype	Case (number)	Control (number)	Before adjusted		After adjusted		Power
					OR (95%CI)	***p***^a^	OR (95%CI)	***p***^b^	
rs12482371	Genotype	TT	217	208	1.00		1.00		68.97%
		CT	185	171	1.04 (0.78 - 1.38)	0.801	1.04 (0.78 - 1.37)	0.801	
		CC	30	51	**0.56 (0.35** - **0.92)**	0.022^*^	**0.56 (0.35** - **0.92)**	0.022^*^	
	Dominant	TT	217	208	1.00		1.00		
		CT-CC	215	222	0.93 (0.71 - 1.21)	0.585	0.93 (0.71 - 1.21)	0.586	
	Recessive	TT-CT	402	379	1.00		1.00		
		CC	30	51	**0.55 (0.35** - **0.89)**	0.014^*^	**0.55 (0.35** - **0.89)**	0.015^*^	
	Log-additive	–	–	–	0.85 (0.70 - 1.05)	0.129	0.85 (0.70 - 1.05)	0.129	
rs1893650	Genotype	TT	281	279	1.00		1.00		63.95%
		CT	145	135	1.07 (0.80 - 1.42)	0.661	1.07 (0.80 - 1.42)	0.663	
		CC	6	17	**0.35 (0.14** - **0.90)**	0.030^*^	**0.35 (0.14** - **0.90)**	0.030^*^	
	Dominant	TT	281	279	1.00		1.00		
		CT-CC	151	152	0.99 (0.75 - 1.30)	0.923	0.99 (0.75 - 1.3)	0.922	
	Recessive	TT-CT	426	414	1.00		1.00		
		CC	6	17	**0.34 (0.13** - **0.88)**	0.026^*^	**0.34 (0.13** - **0.88)**	0.026^*^	
	Log-additive	–	–	–	0.91 (0.71 - 1.16)	0.433	0.90 (0.71 - 1.16)	0.432	
rs928883	Genotype	GG	90	129	1.00		1.00		97.12%
		AG	244	204	**1.71 (1.24** - **2.38)**	0.001^*^	**1.71 (1.24** - **2.38)**	0.001^*^	
		AA	85	90	1.35 (0.91 - 2.02)	0.138	1.35 (0.9 - 2.02)	0.141	
	Dominant	GG	90	129	1.00		1.00		
		AG-AA	329	294	**1.60 (1.17** - **2.19)**	0.003^*^	**1.60 (1.17** - **2.19)**	0.003^*^	
	Recessive	GG-AG	334	333	1.00		1.00		
		AA	85	90	0.94 (0.67 - 1.31)	0.723	0.94 (0.67 - 1.31)	0.716	
	Log-additive	–	–	–	1.19 (0.97 - 1.45)	0.088	1.19 (0.97 - 1.45)	0.090	

*OR* odds ratio, *SNP* single-nucleotide polymorphism, *95% CI* 95% confidence interval. ^a^*p* values were calculated from unconditional logistic regression analysis. ^b^*p* values were adjusted by age and gender

*p < 0.05 indicates statistical significance

*p < 0.0002 (0.05/8/4) indicates statistical significance for Bonferroni correction

Statistical power of rs12482371, rs1893650, rs928883 was estimated through the online software (http://sampsize.sourceforge.net/iface/s3.html#ccp), which were related to liver cancer risk in our research. The powers for rs12482371, rs1893650, rs928883 were 68.97, 63.95, and 97.12%, achieved a certain statistical power.

### Stratification analysis of age and sex

Next, stratification analysis by age or gender was carried out to evaluate the influence of these candidate SNPs on liver cancer risk (Table 4). The stratification analysis of sex revealed the associations between three SNPs and Liver cancer risk. Two loci (rs12482371 and rs1893650) decreasing risk of Liver cancer in male under genotype model (rs12482371, CC vs T/T, OR = 0.51, *p* = 0.016; rs1893650, CC vs TT, OR = 0.32, *p* = 0.030) and recessive model (rs12482371, C/C vs T/T-C/T, OR = 0.52, *p* = 0.018; rs1893650, CC vs T/T-C/T, OR = 0.32, *p* = 0.030). Rs928883 was observed to increase Liver cancer risk under genotype model (AG vs GG, OR = 1.95, *p* = 0.000) and dominant model (AG-AA vs GG, OR = 1.74, *p* = 0.002). No statistical correlations were found in female in any genetic model.

**Table 4:** The relationship of three gene polymorphisms with liver cancer according to the stratification by gender and age

SNP	Model	Genotype	Male		Female		> 55		≤55	
			OR (95%CI)	*p*	OR (95%CI)	*p*	OR (95%CI)	*p*	OR (95%CI)	*p*
rs12482371	Allele	T	1.00		1.00		1.00		1.00	
		C	0.79 (0.63 - 1.00)	0.046	1.11 (0.71 - 1.76)	0.642	0.88 (0.65 - 1.19)	0.410	0.81 (0.61 - 1.08)	0.155
	Genotype	TT	1.00		1.00		1.00		1.00	
		CT	0.93 (0.68 - 1.28)	0.651	1.55 (0.83 - 2.92)	0.170	1.08 (0.71 - 1.65)	0.718	0.97 (0.66 - 1.43)	0.895
		CC	**0.51 (0.29** - **0.88)**	0.016^*^	0.82 (0.29 - 2.36)	0.716	0.60 (0.30 - 1.21)	0.156	0.52 (0.26 - 1.04)	0.063
	Dominant	TT	1.00		1.00		1.00		1.00	
		CT-CC	0.83 (0.62 - 1.13)	0.236	1.38 (0.76 - 2.49)	0.293	0.97 (0.65 - 1.44)	0.871	0.87 (0.60 - 1.26)	0.460
	Recessive	TT-CT	1.00		1.00		1.00		1.00	
		CC	**0.52 (0.31** - **0.90)**	0.018^*^	0.67 (0.24 - 1.86)	0.448	0.58 (0.30 - 1.14)	0.112	0.52 (0.27 - 1.03)	0.059
	Log-additive	–	0.79 (0.63 - 1.00)	0.049	1.11 (0.71 - 1.74)	0.648	0.88 (0.65 - 1.19)	0.395	0.82 (0.62 - 1.08)	0.161
rs1893650	Allele	T	1.00		1.00		1.00		1.00	
		C	0.85 (0.65 - 1.11)	0.224	1.22 (0.70 - 2.12)	0.481	1.01 (0.71 - 1.44)	0.956	0.82 (0.59 - 1.15)	0.252
	Genotype	TT	1.00		1.00		1.00		1.00	
		CT	0.99 (0.71 - 1.36)	0.929	1.43 (0.75 - 2.72)	0.279	1.13 (0.74 - 1.73)	0.577	1.00 (0.67 - 1.48)	0.984
		CC	**0.32 (0.11** - **0.90)**	0.030^*^	0.55 (0.05 - 6.25)	0.627	0.66 (0.20 - 2.14)	0.491	**0.10 (0.01** - **0.80)**	0.030*
	Dominant	TT	1.00		1.00		1.00		1.00	
		CT-CC	0.91 (0.66 - 1.24)	0.537	1.36 (0.72 - 2.56)	0.339	1.08 (0.71 - 1.63)	0.719	0.89 (0.61 - 1.32)	0.570
	Recessive	TT-CT	1.00		1.00		1.00		1.00	
		CC	**0.32 (0.12** - **0.90)**	0.030^*^	0.49 (0.04 - 5.51)	0.560	0.63 (0.20 - 2.04)	0.445	**0.10 (0.01** - **0.79)**	0.029*
	Log-additive	–	0.84 (0.64-1.11)	0.212	1.24 (0.69 - 2.23)	0.464	1.01 (0.71 - 1.46)	0.941	0.80 (0.56 - 1.13)	0.206
rs928883	Allele	G	1.00		1.00		1.00		1.00	
		A	1.17 (0.94 - 1.45)	0.155	1.20 (0.78-1.83)	0.406	1.12 (0.85 - 1.49)	0.420	1.22 (0.94 - 1.59)	0.130
	Genotype	GG	1.00		1.00		1.00		1.00	
		AG	**1.95 (1.34** - **2.82)**	0.000^*^	1.11 (0.55 - 2.23)	0.766	**1.84 (1.14** - **2.98)**	0.013^*^	**1.62 (1.03** - **2.55)**	0.036*
		AA	1.34 (0.86 - 2.09)	0.203	1.54 (0.62 - 3.87)	0.356	1.20 (0.66 - 2.17)	0.547	1.53 (0.88 - 2.65)	0.130
	Dominant	GG	1.00		1.00		1.00		1.00	
		AG-AA	**1.74 (1.23** - **2.48)**	0.002^*^	1.20 (0.62 - 2.34)	0.588	**1.63 (1.03** - **2.58)**	0.036^*^	**1.59 (1.04** - **2.45)**	0.034*
	Recessive	GG-AG	1.00		1.00		1.00		1.00	
		AA	0.85 (0.59 - 1.23)	0.399	1.44 (0.65 - 3.19)	0.370	0.80 (0.49 - 1.31)	0.373	1.10 (0.70 - 1.74)	0.671
	Log-additive	–	1.18 (0.95 - 1.47)	0.144	1.22 (0.78 - 1.92)	0.381	1.13 (0.84 - 1.52)	0.408	1.25 (0.95 - 1.65)	0.106

*OR* odds ratio, *95%CI* 95% confidence interval

Bold indicates statistical significance (*p* < 0.05)

*p < 0.00625 (0.05/8) indicates statistical significance for Bonferroni correction

Furthermore, we conducted the stratification analysis of age. At age > 55 years group and age ≤ 55 years group, we found that *MIR155HG* rs928883 added Liver cancer risk under genotype model (AG vs GG: age > 55 years group: OR = 1.84, *p* = 0.013; age ≤ 55 years group: OR = 1.62, *p* = 0.036) and dominant model (AG-AA vs GG: age > 55 years group: OR = 1.63, *p* = 0.036; age ≤ 55 years group: OR = 1.59, *p* = 0.034). For *MIR155HG* rs1893650, we only found that *MIR155HG* rs1893650 reduced Liver cancer risk in age ≤ 55 years under genotype model (CC vs TT, OR = 0.10, *p* = 0.030) and recessive model (CC vs TT-CT, OR = 0.10, *p* = 0.029).

### Haplotype analyses

**Fig. 1:**
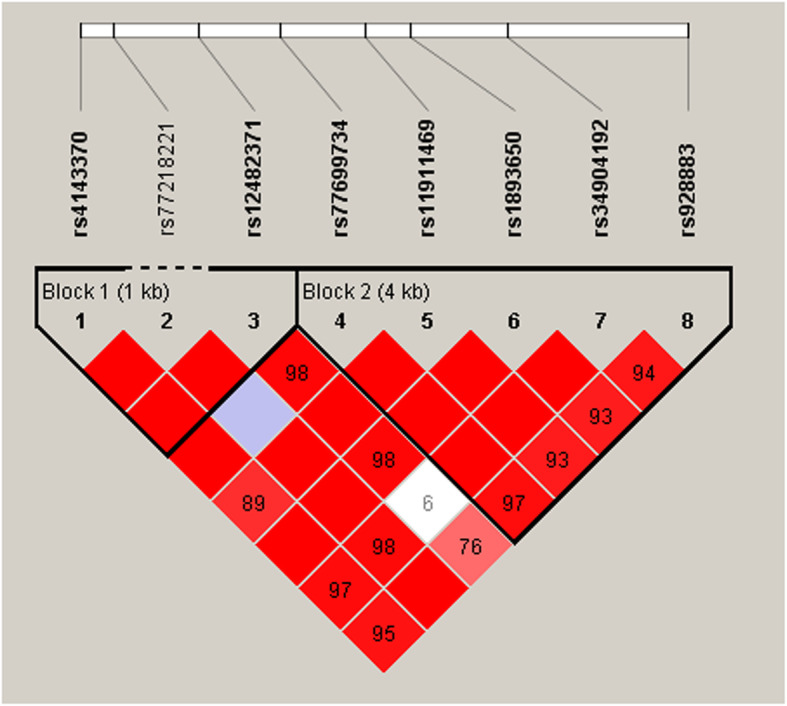
Linkage disequilibrium (LD) plots containing SNPs from *MIR155HG*

LD construction and haplotype analyses of these SNPs were performed in cases and controls. All SNPs existed in two LD blocks (Block 1: rs4143370 and rs12482371; Block 2: rs77699734, rs11911469, rs1893650, rs34904192 and rs928883) in *MIR155HG* (Fig. 1). Haplotype “GATAG” in Block 2 increased the risk of liver cancer (OR = 1.44, *p* = 0.047) (Table 5). However, we didn’t find a significant correlation between the other haplotypes and Liver cancer risk.

**Table 5:** Haplotype analysis results in this study

Block	Haplotype	Frequency		OR (95%CI)	*p*
		Case	Control		
Block 1	rs4143370|rs12482371				
	GT	0.284	0.317	0.85 (0.70 - 1.05)	0.129
	CC	0.831	0.845	0.9 (0.70 - 1.16)	0.424
	GC	0.548	0.528	1.08 (0.90 - 1.31)	0.414
Block 2	rs77699734|rs11911469|rs1893650|rs34904192|rs928883				
	CATGA	0.527	0.554	0.9 (0.74 - 1.09)	0.267
	GATAG	0.932	0.905	1.44 (1.01 - 2.06)	0.047^*^
	CATAG	0.850	0.849	1.01 (0.78 - 1.32)	0.932
	CACGG	0.172	0.190	0.87 (0.68 - 1.13)	0.311
	CCTGG	0.116	0.106	1.12 (0.82 - 1.52)	0.477

*OR* odds ratio, *pa* adjusted by gender and age, *SNP* single nucleotide polymorphism, *95% CI* 95% confidence interval

Bold indicates statistical significance (*p* < 0.05)

## Discussion

In this study, we assessed the associations of selected SNPs in *MIR155HG* with liver cancer susceptibility in the Han Chinese population. We demonstrated that *MIR155HG* genetic polymorphisms are correlated with liver cancer risk among Han Chinese individuals. Our findings uncovered that rs12482371 and rs1893650 in *MIR155HG* were related to reduce Liver cancer risk, and rs928883 increased Liver cancer risk. Furthermore, apart from the rs77218221, other selected SNPs could form two LD blocks. Haplotype “GATAG” in Block 2 was found to elevate the liver cancer risk. These results indicated the importance of *MIR155HG* gene polymorphism in liver cancer development among the Han Chinese population.

Non-coding RNAs are non-protein encoding transcripts closely related to gene transcription, epigenetic regulation and protein translation, which include miRNAs, piwiRNAs and long non-coding RNAs (lncRNAs). Previous studies have confirmed the functional interaction between lncRNA and miRNA, as well as the fact that some lncRNAs themselves may encode miRNAs [24]. The dis-regulation of miR-155 can affect cancer development [25]. The increased expression of miR-155 accompanied by the occurrence of several cancers such as glioma, breast cancer, lung cancer and gastric cancer has been established recently [26, 27]. Therefore, targeting miR-155 is considered a promising method for the treatment of hematopoietic and solid tumors. *MIR155HG* was the primary micro (mi)RNA of miR-155 [18]. However, underlying mechanisms of host miR-155 in modulating tumor growth are still poorly understood. Xuechao Wu et al. demonstrated that *MIR155HG* was an independent adverse prognostic factor in glioma. And its repression inhibited glioma growth in vitro and in vivo [19]. Veerakumar Balasubramaniyan et al. raised that targeting *MIR155HG* can be a novel approach in glioma [28]. And miR-155 is a transcriptional product of its host gene (*MIR155HG*), the genetic variation of *MIR155HG* gene and *miR-155* gene may affected its expression [29].

To date, however, there were no studies focusing on the influence of *MIR155HG* genetic variations on liver cancer risk. Our findings suggest that our hypothesis that *MIR155HG* polymorphisms were related to liver cancer risk in Chinese is correct. Our results showed that rs12482371 and rs1893650 in the *MIR155HG* were associated with protection from Liver cancer. And the rs928883 was connected with an added risk of Liver cancer. One research found that the allele “C” of rs12482371 and rs1893650, and the allele “A” of rs928883 in *MIR155HG* were correlated to colorectal cancer risk [30]. Moreover, the sites selected in this study are existed in the intron region of *MIR155HG*. This is consistent with our recent understanding of introns. Introns are no longer what we traditionally think of as meaningless. Studies have shown that it can influence transcription level by affecting transcription rate, nuclear output and transcription stability, and also can improve the efficiency of mRNA translation [31]. Although the specific mechanism of rs12482371, rs1893650, and rs928883 on the *MIR155HG* affecting liver cancer is unclear, it is worthwhile to reveal the correlation between the SNPs and liver cancer susceptibility. Meanwhile, our results will be helpful for the prevention and early detection of Liver cancer and give theoretical basis.

There are several limitations to consider in the current study. Our research is fundamental, and it does not performed experiments to investigate the expression of miR-155 and its predicted targets. So, the exact molecular mechanism by which SNP alters the expression of *MIR155HG* needs to be clarified in future work.

## Conclusions

To sum up, our research first demonstrated that *MIR155HG* gene polymorphisms were correlated with the susceptibility to Liver cancer among Han Chinese population, in particular males and age ≤ 55 years old.

## Supplementary information

**Additional file 1: Table 1.** PCR primer for this study

**Additional file 2: Table 2.** the Genetic polymorphisms of eight locus in 432 liver cancer cases and 430 healthy controls

## Data Availability

All data generated or analysed during this study are included in this published article and its supplementary information files.

## Abbreviations

SNPs: Single nucleotide polymorphisms
miRNA-155 HG: miRNA-155 host gene
OR: Odds ratio
CI: Confidence interval
MAF: Minor allele frequency
EDTA: Ethylenediamine tetraacetic acid
HWE: Hardy–Weinberg equilibrium
LD: Linkage disequilibrium
HCC: Hepatocellular carcinoma
HBV: Hepatitis B virus
LncRNAs: Long noncoding RNAs
