# Supplementary material for: MiRNA155HG polymorphisms influenced the risk of liver cancer among the Han Chinese population
Source: BMC Med Genet. 2020 Jun 19;21:134. doi: 10.1186/s12881-020-01064-4 (PMC7304092; doi:10.1186/s12881-020-01064-4)
Supplement: Supplementary file 1 — Additional file 1: Table 1. PCR primer for this study [file 12881_2020_1064_MOESM1_ESM.docx]

**Supplementary Table 1. PCR primer for this study.**

| SNP-ID | Foward primer (5'-3') for PCR | Reverse primer (5'-3') for PCR | UEP SEQ |
| --- | --- | --- | --- |
| rs4143370 | ACGTTGGATGGCTTCAGCTAAGCAATAGCC | ACGTTGGATGTCTATCACTTGGACTGGGC | gtcaaTTGGACTGGGCACCAGCTA |
| rs77218221 | ACGTTGGATGTGAGACACAGCTGGTCTTAG | ACGTTGGATGTGAGTCCAGTAAGATCAGTG | ggaggAAGATCAGTGAAAGACAAAA |
| rs12482371 | ACGTTGGATGATGAGTATTCACACCCTCCC | ACGTTGGATGATGAGTATTCACACCCTCCC | ccaaCTGTGCCTCAAGGCCCCTG |
| rs77699734 | ACGTTGGATGTTAGGAGATCCGTGCTCAAG | ACGTTGGATGCTCTCTGTATCCCTACCCAT | atatcTGCAAACCTGGCATCTAACTT |
| rs11911469 | ACGTTGGATGGCCATTATGGTGTGTGGAAG | ACGTTGGATGCCTGTTTGTTTTTTGTATCCG | TGCCTCTATCATAGGAAAAAATTGC |
| rs1893650 | ACGTTGGATGGTGCTTTCGCTTTTCTGATG | ACGTTGGATGCCTGCTTCTGCTTATTCCAC | cCTGCTTATTCCACAGAATGAG |
| rs34904192 | ACGTTGGATGGAAAGATAAGGATGTACCCC | ACGTTGGATGGTTGCTGCTTTATGCTGGAC | ccgagGCTGGACATGAAAAGTGT |
| rs928883 | ACGTTGGATGGACAGTGCTAACCAAGTAAG | ACGTTGGATGACTACCTAGTCAAAGACCCC | ctACCCCAGTCTAATTTCTAAT |

SNP, Single-nucleotide polymorphism; UEP SEQ, Unextended mini-sequencing primer.
